# Broadband Mechanically Tunable Metasurface Reflectivity Modulator in the Visible Spectrum

**DOI:** 10.1021/acsphotonics.3c00276

**Published:** 2023-05-31

**Authors:** Dorian Herle, Andrei Kiselev, Luis Guillermo Villanueva, Olivier J. F. Martin, Niels Quack

**Affiliations:** † Ecole Polytechnique Federale de Lausanne, Advanced Nano-Mechanical Systems Laboratory, EPFL STI IGM NEMS, Station 9, CH-1015 Lausanne, Switzerland; ‡ Ecole Polytechnique Federale de Lausanne, Nanophotonics and Metrology Laboratory, EPFL STI IMT NAM, Station 11, CH-1015 Lausanne, Switzerland; § School of Aerospace, Mechanical and Mechatronic Engineering, Mechanical Engineering (J07), University of Sydney, Blackwattle Creek Ln, Darlington, NSW 2008, Australia

**Keywords:** metasurface, MEMS, optics, tunability, visible spectrum

## Abstract

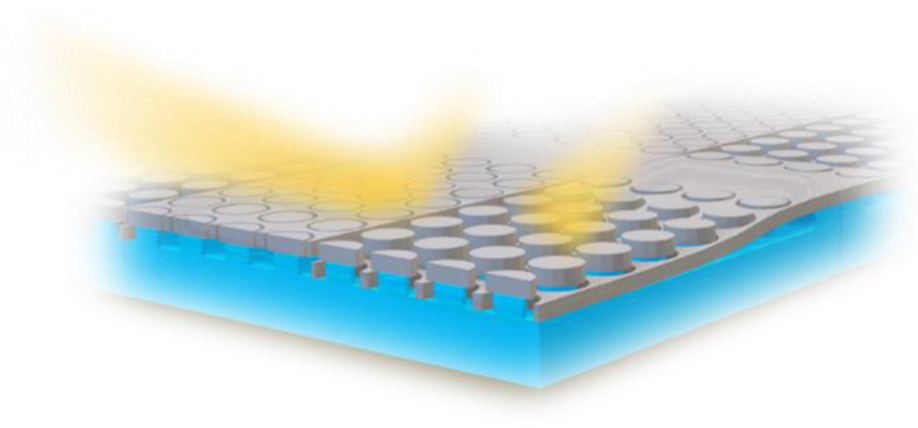

Reflectivity modulation is a critical feature for applications in telecommunications, 3D imaging and printing, advanced laser machining, or portable displays. Tunable metasurfaces have recently emerged as a promising implementation for miniaturized and high-performance tunable optical components. Commonly, metasurface response tuning is achieved by electro-optical effects. In this work, we demonstrate reflectivity modulation based on a nanostructured, mechanically tunable, metasurface, consisting of an amorphous silicon nanopillar array and a suspended amorphous silicon membrane with integrated electrostatic actuators. With a membrane displacement of only 150 nm, we demonstrate reflectivity modulation by Mie resonance enhanced absorption in the pillar array, leading to a reflectivity contrast ratio of 1:3 over the spectral range from 400–530 nm. With fast, low-power electrostatic actuation and a broadband response in the visible spectrum, this mechanically tunable metasurface reflectivity modulator could enable high frame rate dynamic reflective displays.

## Introduction

Optical metasurfaces are material composites consisting of subwavelength structures that have been engineered to exhibit specific electromagnetic properties, which typically cannot be achieved with a bulk material alone. As such, metasurfaces have enabled original developments in a variety of applications. Recent demonstrations include, for instance, low cost and robust THz bandpass filters^1^ for medical uses and security screening, enhancing short-wave infrared imagers with complementary metal-oxide-semiconductor compatible polarization filters^2^ to improve the inspection of materials, printing images with a resolution of more than 100,000 dpi,^3^ femtomolar bio-sensing for antibody and antigen detection,^4^ flat metasurface lenses for high-resolution optical microscope imaging^5^ at the diffraction-limit, and holograms for security printing on curved substrates.^6^ Metasurfaces offer a high degree of scalability and integration, which is continuously improving, due to the advancements in micro- and nanofabrication technologies. While the cited demonstrations have hitherto primarily been built on passive building-blocks, advancements in active or tunable components are opening avenues to new and exciting research opportunities and industrial applications,^7^ such as optical beam steering^8^ for LIDAR and laser machining, or reflective displays^9^ for outdoor signage or mobile applications. Metasurfaces induce changes in the electromagnetic response both through material and geometric properties; thus, active modulation methods focus on either of these.^10^ For example, changes of the material refractive index can be introduced through phase change,^11−13^ liquid crystal,^14^ or non-linear materials.^15^ At longer wavelength, it is also common to find the integration of PIN-diodes^16^ or varactors^17^ to actively control induced currents. On the other hand, geometric reconfiguration can be achieved by stretching a flexible substrate,^18^ displacing multi-layered metasurfaces,^19^ or generally by micro-electromechanical systems (MEMS)^20^ based technology. The latter is promising for metasurface tuning as geometric reconfigurations can induce large changes in the local field and consequently offer a large dynamic range in the electromagnetic response. In addition, MEMS actuation methods typically offer low power consumption, fast reconfiguration at MHz rates, and high compatibility with industrial fabrication processes, and have opened up the possibility to create a wide variety of movable and tunable optical structures.^21^ A majority of MEMS tunable metasurfaces have been demonstrated in the near infrared and THz for applications in telecommunications or imaging, such as MEMS based tunable flat lenses for varifocal components^22^ or tunable THz absorbers.^23^ In this work, we make use of the advances in fabrication technologies to achieve MEMS that operate a tunable metasurface in the visible domain. As these types of devices require stringent control of geometric dimensions and the associated lithography and nanofabrication processes are challenging, the majority of research on tunable metasurfaces in the visible spectrum has hitherto been carried out on electro-optical materials and focused on introducing a shift in the spectral response.^24,25^ Less attention has been given to the tuning of a broadband amplitude response. In our work, we overcome these challenges and experimentally demonstrate a MEMS tunable metasurface for broadband tunable reflective modulation, with potential applications in reflective or projection displays.

## Results and Discussion

### Working Principle

**Figure 1:**
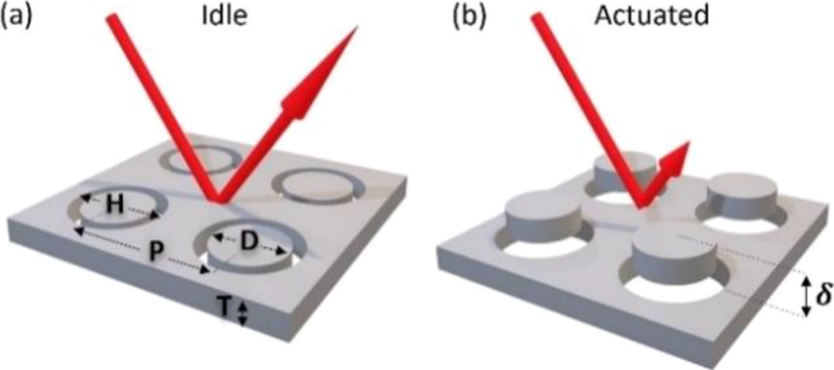
Working principle of the mechanically tunable metasurface reflectivity modulator, consisting of a movable membrane with disk-filled holes. (a) In the idle state, the membrane top surface and disk top surface are aligned, leading to a highly reflective surface. (b) In the actuated state, the membrane is vertically displaced by a distance δ, and Mie resonance-induced absorption in the nano-disks leads to low reflectivity.

The working principle of our MEMS tunable metasurface is based on tunable light absorption by displacement of a nanostructured membrane to change the broadband amplitude reflectivity in the visible. Static light absorption enhancement using micro- and nano-structures has been well-studied for applications in solar-cells to improve their efficiency^26^ by light-trapping. Such sub-wavelength structures are commonly designed as silicon nano-disks, motivated by the ease of manufacturing, due to the abundance of silicon as a raw material and established foundry processes. Highly efficient light trapping structures have been shown to reduce reflectivity to as low as 1.3%,^27^ which is also referred to as *black silicon*. The enhanced absorption has been demonstrated to be caused by the effective excitation of resonances in the system of periodically displaced silicon pillars.^28^ In this work, we use a movable membrane as an effective background medium to control the Mie resonance-induced absorption in an array of amorphous silicon (aSi) disks. The working principle of our MEMS tunable metasurface is visualized in Figure 1.

The metasurface acts as a reflective surface in the idle state, which can be turned into a nano-structured surface exhibiting enhanced absorption and consequently reduced reflectivity in the actuated state. The reflectivity modulation is achieved by MEMS actuation of a vertically movable membrane that is structured with a regular hole pattern, with each hole surrounding a fixed nano-disk. A vertical displacement δ between the membrane and the disks allows tuning the surface geometry. In the idle state, the membrane top surface and the disk top surface are aligned, providing high reflectivity. In the actuated state, the membrane is vertically displaced along the axes of the disks, and the light absorption inside the disks is increased, while the reflectivity of the surface reduced. The reflectivity in the idle state of the metasurface is slightly lower compared to a completely flat surface due to scattering losses introduced by the nanosized annular apertures required for the vertical membrane displacement,^29^ yet high contrast ratios (CRs) can still be achieved with this method. Amorphous silicon was selected as the MEMS material for our metasurface due to its notable absorption in the visible range, which is required to achieve low reflectivity in the actuated state, and its compatibility with well-established standard microfabrication processes essential for ensuring scalability. It is important to note that employing aSi for both the disks and the membrane results in increased absorption in the idle state, subsequently leading to a reduced CR. While utilizing distinct materials for the membrane and disks could potentially improve the CR, this consideration lies beyond the scope of this paper as our primary objective was to develop a scalable proof-of-concept using accessible fabrication techniques.

We perform Rigorous Coupled Wave Analysis (RCWA) to simulate the optical performance of our metasurface in both the idle- and the actuated states. To optimize the CR, a particle-based optimization loop has been implemented in python, taking into account the fabrication constraints of a critical dimension of 20 nm. Details on the simulation procedure and optimization routine are further discussed in the Methods section. We use RCWA to calculate the spectral response of our metasurface in the visible range with the optimized geometry parameters as summarized in Table 1 for membrane displacements varying from 0 to 150 nm.

**Table 1:** Design Parameters

symbol	description	value (nm)
*D*	disk diameter	200
*P*	period of disk array	400
*H*	diameter of hole in membrane	300
*T*	thickness of membrane and disk	100
δ	membrane vertical displacement	0 (idle)–150 (actuated)

**Figure 2:**
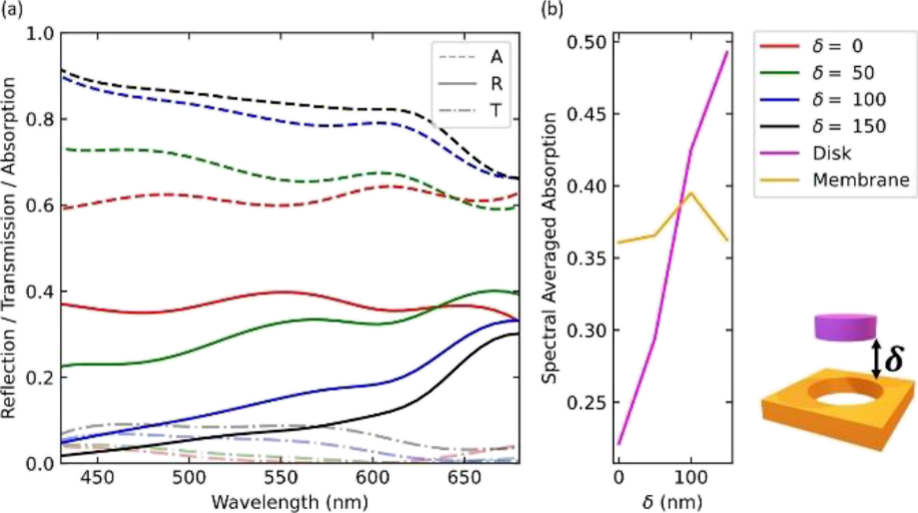
Influence of the membrane position on the optical spectral response. (a) 0th order reflection and overall absorption computed for different membrane positions. A vertical membrane displacement from 0 to 150 nm results in a reflection reduction in the spectral range from 400–650 nm. A: Absorption, R: Reflection, T: Transmission. (b) Spectrally averaged absorption for the disk or the membrane. The disk acts as the active element with a continuous increase in absorption as the membrane is displaced.

Figure 2a depicts the wavelength-dependent reflection, transmission, and absorption of the system. We also calculate the spectral-averaged absorption in disks and membrane separately by integrating and averaging the absorption in each of the components of the system from 400 to 650 nm. With increasing membrane displacement, the spectral-averaged absorption in the nanodisks increases significantly (see Figure 2b), which results in a broadband decrease in reflection from the metasurface.

**Figure 3:**
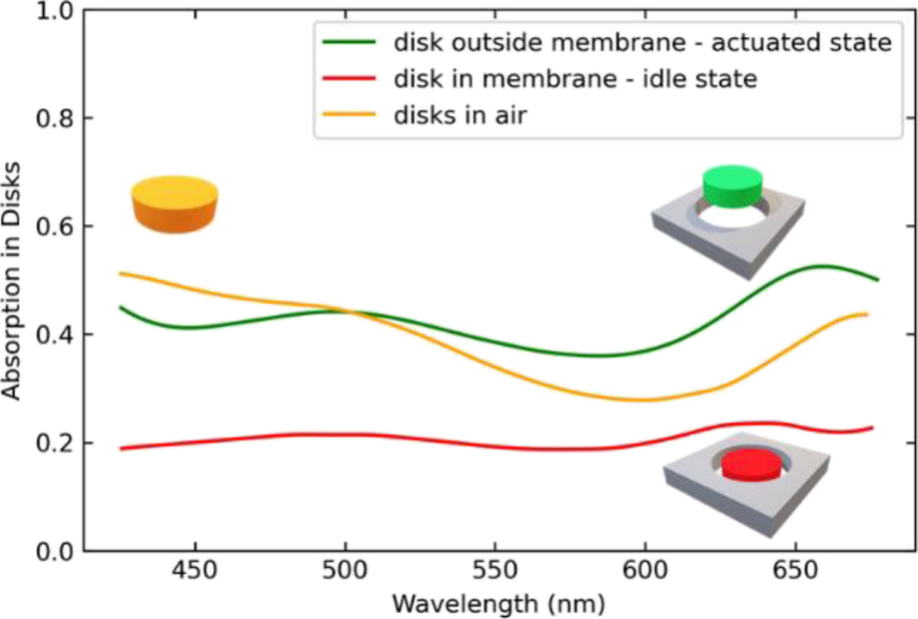
Absorption spectra inside the array of disks freestanding in air, placed in the membrane, and out of the membrane with an offset δ = 150 nm. The parameters for the disks and membrane are the same as in Table 1.

To provide additional insights into the origin of the broadband spectral response, we study the coupling between the membrane and the disks in further detail. We first perform in Figure 3 finite elements simulations to extract the absorption inside the disks, for disks in air without any surrounding membrane, for disks surrounded by a well-aligned membrane, and for disks placed at the vertical distance δ = 150 nm above the membrane. We can see from Figure 3 that disks in air and disks were offset by a distance of 150 nm above the membrane and exhibited similar absorption spectra. The discrepancy between both curves starting mostly from 500 nm can be attributed to coupling between disks and membrane in the latter. This coupling is wavelength dependent, thus explaining the non-constant offset. For disks that are inside the membrane, however, the absorption within the disks decreases. We hypothesize that upon insertion of the disks into the membrane, the membrane serves as an effective medium with high refractive index that prevents an effective excitation of the Mie resonances in the disks. Consequently, we do not observe the resonant absorption when the disks are inside the membrane.

**Figure 4:**
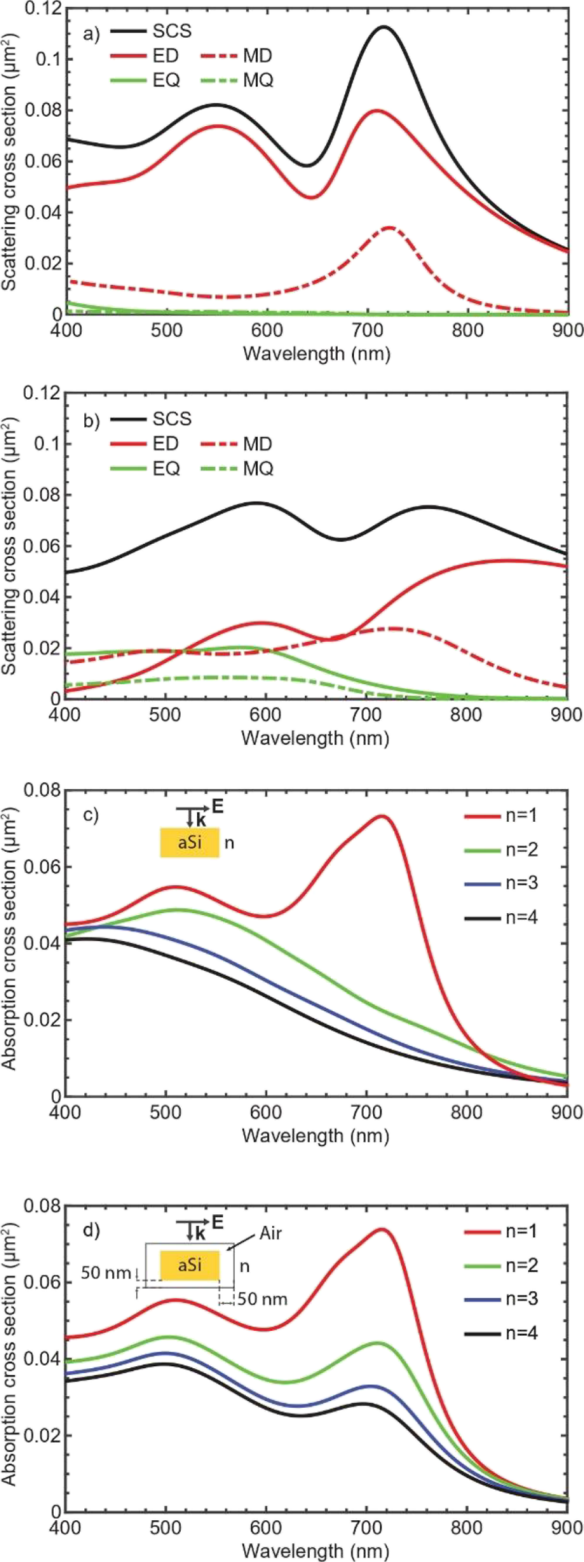
Spectral response of an isolated nano-disk surrounded by a medium with different refractive indices. Scattering cross-section and its multipolar decomposition for an isolated disk in backgrounds (a) *n* = 1 (a) and (b) *n* = 2. (c) Absorption for the isolated disks in different background media from *n* = 1 to *n* = 4. (d) Absorption for an isolated disk placed in an air shell, which is further placed in a medium with the refractive index *n* = 1 to *n* = 4. A resonant behavior is observed for a disk in air (*n* = 1), while the resonances broaden for surrounding media with increased refractive indices. Abbreviations: SCS = Scattering Cross Section, ED = Electric Dipole, EQ = Electric Quadrupole, MD = Magnetic Dipole, MQ = Magnetic Quadrupole.

In order to study this effect, we perform a decomposition of the field scattered by an individual disk into vector spherical harmonics.^30^ The scattering cross-sections along with the multipolar components are shown in Figure 4a,b, for an isolated disk, having the same size as in Figure 2, placed inside a medium with the refractive index of *n* = 1 (Figure 4a) and *n* = 2 (Figure 4b). For the multipolar analysis, it is sufficient to consider these first four multipoles to fully recover the complete scattering cross section. As can be seen from Figure 4a,b, the individual resonances broaden with increasing the refractive index of the host medium. For example, the magnetic dipole of the disk surrounded by a medium of refractive index 2 as shown in Figure 4b exhibits a larger width compared to the disk surrounded by a medium of refractive index 1 as shown in Figure 4a. The same effect takes place for the electric dipole. We also observe that relatively broad higher order multipoles—the electric and magnetic quadrupoles—redshift and appear in the visible part of the spectrum. This resonance broadening leads to a lower field enhancement inside the structure.^31^ In Figure 4c, we study the absorption cross section of an isolated disk and vary the refractive index of the surrounding medium over a broad range to mimic the gradual change of the effective refractive index of the surrounding medium as disks enter the membrane. As can be seen from Figure 4c, this leads to a decrease of the absorption cross-section. However, as can be seen from Figure 4c, the absorption barely changes for the short wavelength part of the spectrum close to 400 nm. We attribute this to the fact that the air spacing between the membrane and the disks was not considered. To include the effect of air spacing, in Figure 4d, we plot the absorption cross section for an isolated disk placed in an air void that is further submerged in a medium with different refractive indices. In this geometry, the air spacing thickness corresponds to the disk-membrane distance measured for the fabricated device. From Figure 4d, it becomes apparent the absorption over the entire range of interest (400–650 nm) drops as the refractive index of the surrounding medium increases. These simulations support our assumption about the nature of the observed absorption increment for the fabricated device. Consequently, we can understand that in the idle state, the membrane can be perceived as a high refractive index host medium that decreases the absorption in the disks. By offsetting the membrane, we thus actively tune the disks host medium and, consequently, the amount of light absorbed inside the disks.

### Device Design

**Figure 5:**
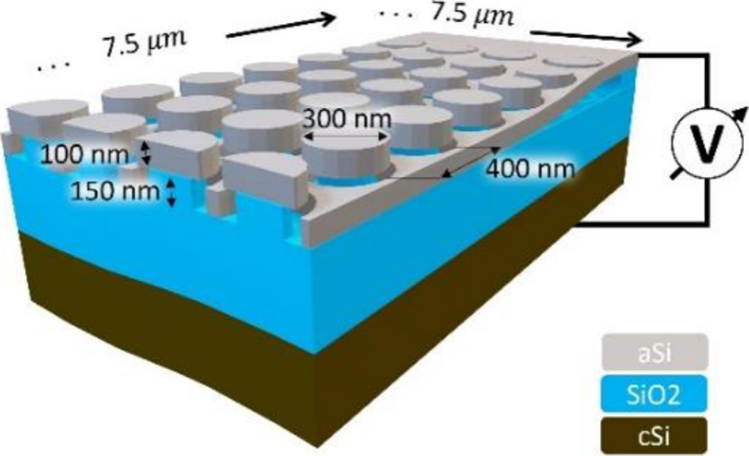
MEMS Design. Oxide supported disks and membrane. Electrostatic actuation of the membrane by the application of a potential between the membrane and the support silicon, whereas oxide acts as a dielectric spacer. Disk height as well as membrane thickness is chosen to be 100 nm, with an oxide stand of 150 nm and a dielectric oxide spacer of 350 nm.

The design of our MEMS actuated metasurface consists of a perforated suspended microbridge where each perforation hosts a nano-pillar. The said pillar consists of an amorphous silicon nano-disk that is supported by a silicon oxide stand. The device is schematically represented in Figure 5. By applying a voltage between membrane and substrate, the membrane can be continuously displaced through electrostatic attraction. In this work, the oxide stands were chosen to be of 150 nm height, allowing the membrane to travel this distance. The device is operated in pull-in, with a pull-in voltage analytically approximated to be on the order of 14–16 V. The derivation can be found in the Supporting Information.

### Fabrication

**Figure 6:**
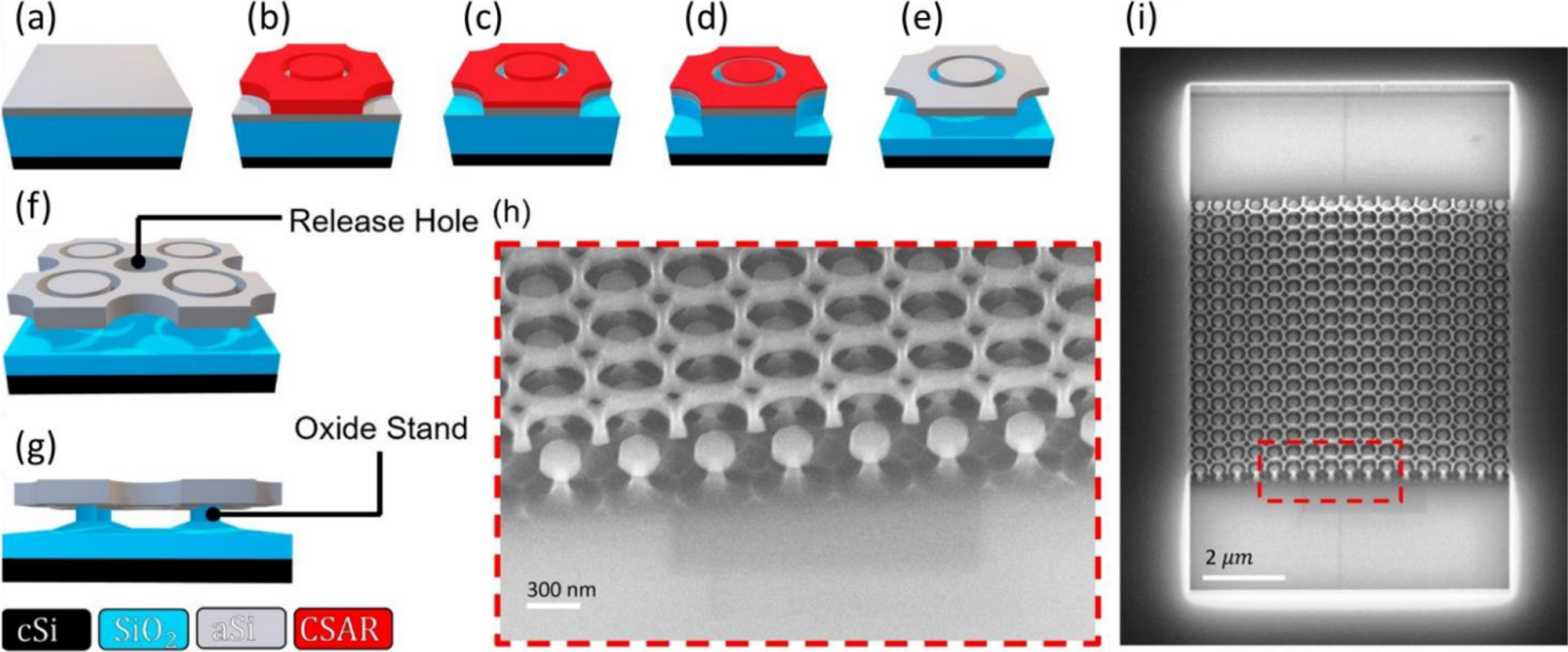
Fabrication of a tunable metasurface. (a–e) Outline of the process flow showing the unit cell of the periodic pattern: (a) deposition of 500 nm of SiO_2_ and 80 nm of amorphous silicon (aSi) onto a crystalline silicon (cSi) wafer, (b) electron-beam lithography using positive resist CSAR 64 of 150 nm thickness, (c) reactive ion etching of the aSi layer, (d) followed by approximately 100 nm reactive ion etching into the SiO_2_, (e) and carefully timed hydrofluoric (HF) vapor phase isotropic SiO_2_ etching to release the membrane. (g) Visualization of the release holes in the membrane, which enable increased access points for the HF vapor to etch the sacrificial SiO_2_ layer, thus releasing the membrane faster than a complete under-etch of the disk. (i) Visualization of the remaining oxide stand after the release step, which keeps the disks in place. (h) and (i) SEM images of the fabricated sample, showcasing (i) a single pixel consisting of a clamped-clamped membrane as well as (h) a close-up showing the individual aSi disks supported by the remaining oxide posts, and the membrane with its holes, slightly buckled upwards (80 nm max) due to residual stress (thermal mismatch during deposition).

**Figure 7:**
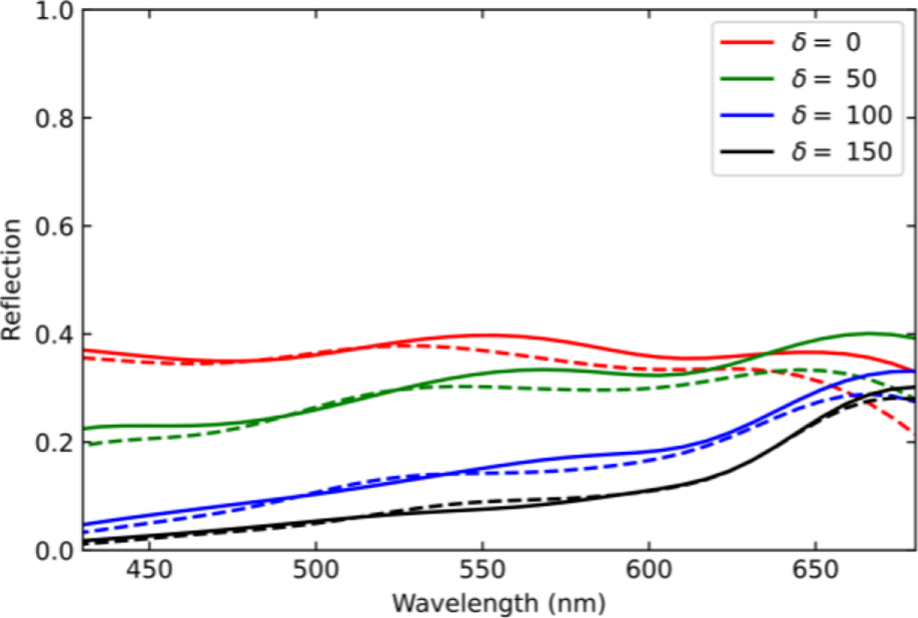
Influence of the release holes on the device reflection. Reflection spectra (400–700 nm) are simulated for a membrane without release holes (solid lines) and with 100 nm diameter release holes (dashed lines), showing very little influence on the spectral response.

We fabricate our MEMS tunable metasurface employing a surface micromachining process as detailed in the Methods section. The microfabrication process is shown schematically in Figure 6a–e. The nano-scale dimensions of the annular trench require high resolution patterning and electron-beam lithography is used to define the structure. First, the metasurface material is sputtered onto a wet-oxidized crystalline silicon wafer with 500 nm SiO_2_ on its surface. Following an e-beam exposure (Figure 6b), the aSi surface is patterned (Figure 6c) using inductively coupled plasma (ICP). The latter is followed by a vertical SiO_2_ ICP etch (Figure 6d). This step serves to control the height of the oxide stand (Figure 6g) and to provide lateral access for the etchant during the HF vapor release step (Figure 6e). The release holes diameters of 100 nm are chosen to be below the cut-off hole diameter for the smallest wavelength in the spectrum of interest, thus they do not noticeably affect the optical performance, as can be observed in Figure 7. We have observed that HF Vapor release can be precisely controlled by carefully timing the HF isotropic etching Our etch rate has shown consistent 0.12 nm/s, thus, once calibrated, the release is a simple time-controlled process. In general, this approach allows for the device to be fabricated using only one single lithography, avoiding any alignment issues.

### Experimental Characterization

**Figure 8:**
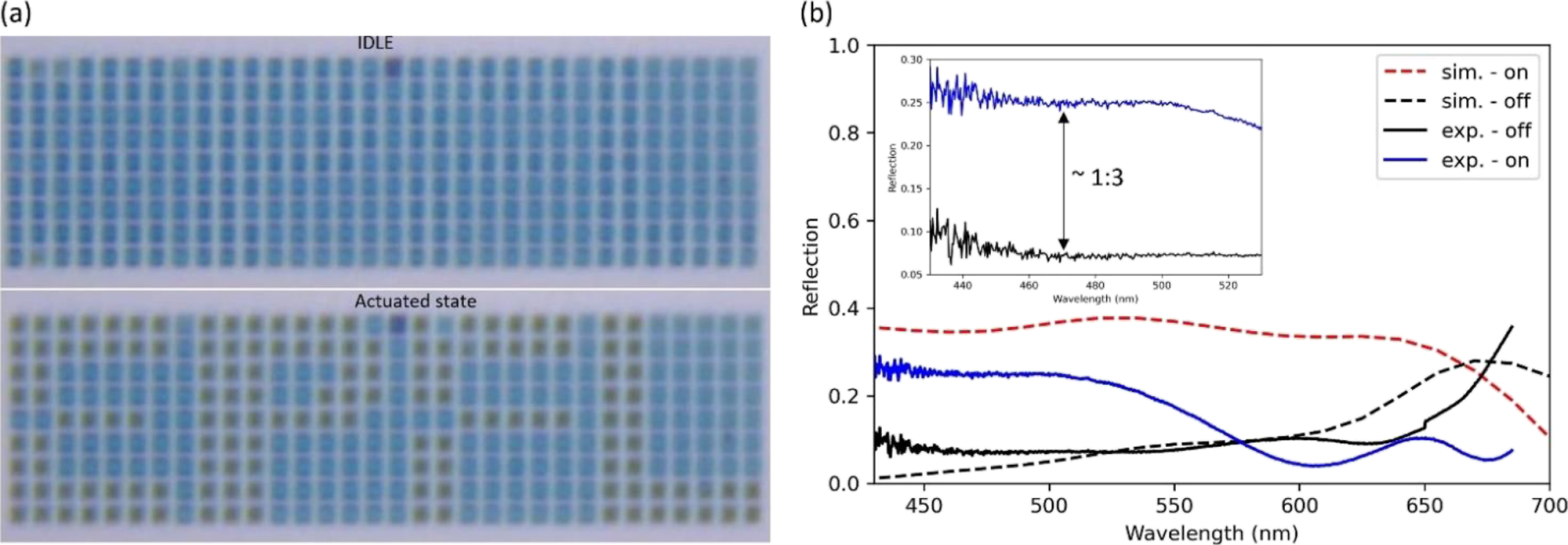
Demonstration of the modulation capability showcasing the EPFL logo when actuated state. (a) Optical microscope image showing a segmented display forming the EPFL logo when switched from the idle to the actuated states. The pixels outside the EPFL logo are electrically disconnected. (b) Measured reflection spectra in the optically idle and actuated states corresponding to applied 0 and 70 V, respectively. The average CR in the spectral range from 400 to 530 nm is 1:3.

**Figure 9:**
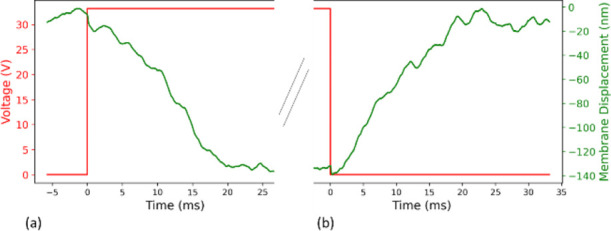
LDV switching time measurement. A step input of 33 Volts induces an averaged membrane displacement of approx. 100 nm. Switching time for both on (a) and off (b) switching ∼20 ms. This slow switching time is due to the high aSi resistivity, leading to a slow RC time constant.

In order to demonstrate the suitability of our tunable metasurface for display applications, we construct a segmented display composed of 42 × 9 individual metasurface pixels according to the design in Figure 6f. All 42 × 9 metasurface pixels have been released and fabricated according to the fabrication process described in the Methods section. Only a subset of pixels was connected electrically to the device layer surface for actuation while the remaining pixels remained disconnected from the device layer surface by electrical insulation trenches. The subset of electrically connected pixels was chosen to display the EPFL logo when actuated, as shown in the microscope recording of Figure 8a (see the Supporting Information for two movies showing the dynamically actuated display). The array is shown in the idle state and in the actuated state, where the pixels are actuated by applying an actuation voltage between the device layer surface and the substrate layer. Upon actuation, the segmented pixels of the EPFL logo switch to a darker absorptive state. No stiction was observed. We attribute this to a reduced contact area due to spikes of sacrificial SiO_2_ resulting from the HF vapor etch undercut of the membrane, as can be observed in the SiO_2_ layer in Figure 6h. We employ a spectrometer setup to experimentally determine the spectral response and CR, using a silver mirror with almost perfect reflectance as reference.^32^Figure 8b shows the measured reflection spectra. We measure an average CR of 1:3 in the range of 400 to 530 nm. The discrepancy to the simulated spectrum in the idle state is due to differences in geometry induced by the manufacturing process. Notably, the membrane is buckled upwards by residual stresses in the idle state. As discussed in further details in the Supplementary Information, this geometry leads to destructive interference in the visible, resulting in a reflection dip between 550 and 700 nm. The membrane can be approximated as an infinite slab of an equivalent refractive index (air holes and aSi). Using this simplified model, it can be seen that for the given membrane thickness destructive interference occurs within the wavelength range of approximately 500 to 700 nm, agreeing with the measured spectra. The switching time is measured to be on the order of 20 ms (see Figure 9), by using laser Doppler velocimetry (LDV) and integrating the result to obtain the membrane displacement versus time. We apply an actuation voltage of 33 V, and we measure a displacement of approximately 140 nm. The actuation voltage was deliberately established at a level beyond the pull-in point to ensure achievement of the actuated state.

The device’s behavior can be regarded as analogous to an RC circuit, wherein the resistance is proportional to the material’s resistivity and the distance between the probe and the pixel. MEMS devices capable of high-frequency modulation typically employ highly doped Si, which can exhibit resistivity as low as 0.001 Ω·cm. In contrast, our experiment utilized amorphous Si (aSi) with a resistivity of 10 kΩ·cm.^33^ Additionally, the experimental probe distance was approximately 1 mm, which would be significantly reduced in a wire-bonded device. The capacity can be approximated as a plate capacitor with an SiO_2_ dielectric spacer of 500 nm and an area of about 1 mm^2^. These values yield an RC time constant of a few tens of microseconds, which concurs well with the measured modulation frequency and switching time. Better optical performances and faster switching can be expected by changing the membrane material to more reflective and more conductive materials, such as aluminum (see Supporting Information for reflection spectra result). The switch off time follows the same speed primarily due to the necessity for the MEMS capacitor to undergo discharge. Consequently, this temporal aspect will also exhibit a strong reliance on the inherent resistances imparted by the membrane material.

## Conclusions

We have demonstrated a tunable MEMS metasurface actuated by the relative displacement of a membrane with holes and an interleaved stationary nano-disk array. We achieve reflectance modulation by introducing a relative offset between the movable membrane and the stationary absorbing nano-disks. Our metasurface is fabricated by surface micromachining using a single e-beam exposure and partial removal of a sacrificial oxide layer that requires no lithography alignment. This prototype exhibits a maximum CR of 1:3 in the visible spectrum from 400 to 530 nm and a switching time of 20 ms, which provides experimental evidence for the suitability of MEMS tunable metasurface reflectance modulators for applications in displays and projection systems.

## Methods

### Microfabrication Process

The metasurface fabrication is carried out at the Center of Micro- and Nanotechnology at EPFL (CMi). A layer of 100 nm of aSi is sputtered (Pfeiffer SPIDER 600, 800 W, 29 sccm O_2_, DC source, room temperature) onto a wet-oxidized (500 nm SiO_2_) crystalline silicon wafer. After a 5 min surface activation step in Plasma Oxygen (500 W, Tepla GiGAbatch), followed by a 5 min dehydration bake at 180 °C, a thin layer (150 nm) of AR-P 6200 (CSAR 62) e-beam resist is spin-coated. This specific e-beam resist is chosen as it is commonly used to define very fine structures, such as 10 nm wide trenches, with a very high contrast. Furthermore, it has a relatively high dry etch selectivity. The resist is pre-exposure baked at 180 °C for 5 min. For the e-beam exposure, a dose of 270 μC/cm^2^ (100 keV, Raith EBPG5000) and proximity-effect correction are applied to minimize the effect of back-scattered electrons, thus optimizing the uniformity across the wafer. Post exposure, the resist is developed for 1 min in amyl-acetate, followed by 1 min in a 90:10 MiBK:IPA rinse solution and dried with nitrogen. The aSi layer is then sub-sequentially etched using ICP (Alcatel AMS 200 SE). A laser-based (λ = 690 nm) end-point detection system is used to precisely stop the etch when reaching the underlying SiO_2_. This is important to prevent unnecessary thinning of the resist as it is still needed as an etch mask for the following directional SiO_2_ etch (Figure 4d). The SiO_2_ etching is also carried out in an ICP-based high density plasma using a mixture of C_4_ F_8_/H_2_/He gases. Finally, the oxide is isotropically etched using HF vapor (SPTS uEtch), yielding the release of the membrane and the creation of the oxide stands. The vapor HF process employs a gas phase comprising anhydrous HF and ethanol (C_2_H_5_OH) at reduced pressure to facilitate the etching of sacrificial SiO_2_ in a vacuum-based system, thereby allowing for the stiction free release of MEMS without the generation of pollutants. To accurately measure the etch-front, high voltage (10 kV) SEM images are taken after specific etching times, allowing a clear view through the aSi layer onto the oxide, thus enabling rapid and very precise oxide etch-rate measurements, yielding 0.12 nm/s.

#### Multipoles Decomposition

The multipolar decomposition is computed using the vector spherical harmonic functions.^30^ The basic idea is that the far field is calculated with the surface integral equation method for isolated structures^34,35^ at the points of a sphere with the radius of 10 microns. Then, this field is decomposed into a series of vector spherical harmonic functions. By knowing the amplitude of these functions, we deduce the scattering cross section attributed to each multipole.^36,30^ The data for the refractive index of aSi were taken from Pierce et al.^37^

#### Optical Design

Particle swarm optimization (PSO) is used to predict the optimal parameters for a maximum spectral averaged contrast between both idle and activated states. A commercial RCWA code^38^ is interfaced through a home-written python wrapper.^39^ The PSO algorithm is implemented utilizing the Python package pyswarms.^40^ The optimization loop works by iteratively simulating new geometries, of which the parameters are defined by the PSO algorithm. As optimization function, the RGB difference between true black for the actuated-state and perfect white for the idle state is used. The RCWA outputs a reflection spectrum (sum of all reflection orders). The reflection spectra are converted into the perceived RGB color using the 1931 ICI Standard Observer convention. The conversion is implemented in the code by utilizing the python package Color.^41^ Furthermore, fabrication constraints such as maximum etch-length to avoid collapse of the nano-disks or minimum achievable gap are all taken into account by adding those as constraints into the PSO algorithm.

## Supporting Information Available

Sinusoidal voltage at 1 Hz (MP4)
Sinusoidal voltage at 100 mHz (MP4)
Derivation of the Pull-In voltage estimation, the mechanical response time, the optical explanation for the reduced contrast between 550 and 700 nm, and a graph showing the effect on the contrast ratio of aluminum as a membrane material (PDF)
