# Supplementary material for: Broadband Mechanically Tunable Metasurface Reflectivity Modulator in the Visible Spectrum
Source: ACS Photonics. 2023 May 31;10(6):1882–9. doi: 10.1021/acsphotonics.3c00276 (PMC10288533; doi:10.1021/acsphotonics.3c00276)
Supplement: Supplementary file 3 — ph3c00276_si_003.pdf [file ph3c00276_si_003.pdf]

Broadband Mechanically Tunable Metasurface Reflectivity Modulator in the Visible

Supporting Information

Dorian Herle[1], Andrei Kiselev[2], Luis Guillermo Villanueva[1], Olivier J.F. Martin[2], and Niels Quack[1,3,**]*

[1] Ecole Polytechnique Federale de Lausanne, Advanced Nano-Mechanical Systems Laboratory
EPFL STI IGM NEMS, Station 9, CH-1015 Lausanne, Switzerland
[2]Ecole Polytechnique Federale de Lausanne, Nanophotonics and Metrology Laboratory
EPFL STI IMT NAM, Station 11, CH-1015 Lausanne, Switzerland
[3]University of Sydney, School of Aerospace, Mechanical and Mechatronic Engineering
Mechanical Engineering (J07),Blackwattle Creek Ln, Darlington NSW 2008, Australia

**Corresponding authors: Dorian Herle, dorian.herle@epfl.ch, Niels Quack, niels.quack@sydney.edu.au*

*** The work reported in this manuscript was performed while N. Quack was at EPFL (affiliation 1), and the manuscript was partially composed while N. Quack was at the University of Sydney (affiliation 3).*

MEMS – Pull-in voltage estimation.

(a)

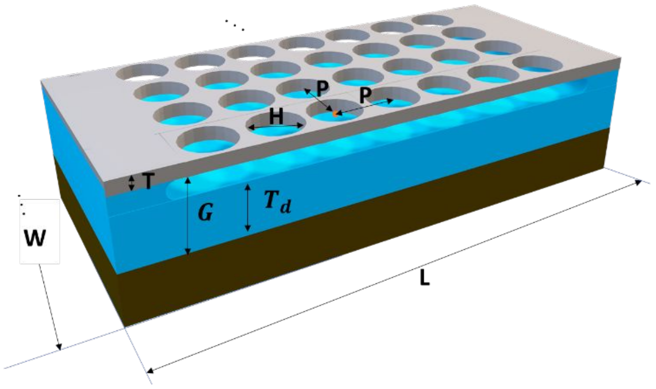

(b)

Geometry		Material	
Name	*Value*	*Name*	*Value*
Hole Diameter *H*	300 nm	Young's Modulus of Amorphous Silicon *E*	80 GPa
Hole Period *P*	400 nm	Relative permittivity of the Dielectric ϵ_{r_D}	3.7
Pixel Width *W*	7.5 µm		
Pixel Length *L*	7.5 µm		
Membrane Thickness *T*	100 nm		
Oxide Thickness T_d	350 nm		
Electrode Distance *G*	500 nm		

Figure S1 Visualization (a) and values of microbridge (b) used for pull-in voltage estimation. Disks are omitted to simplify computation.

A microbridge MEMS design is chosen, where the membrane is clamped on both ends and deflects downwards when a voltage is applied between the membrane with holes and the substrate.

In the following derivation of the pull-in voltage estimation, the fringing fields are neglected. The analytical model used is that of a parallel plate capacitor with movable top plate and with two different dielectric layers (air and oxide) in series between the plates (see Figure S2). The derivation is outlined in the works of Saucedo-Flores et al.[1] and adapted to the present device.

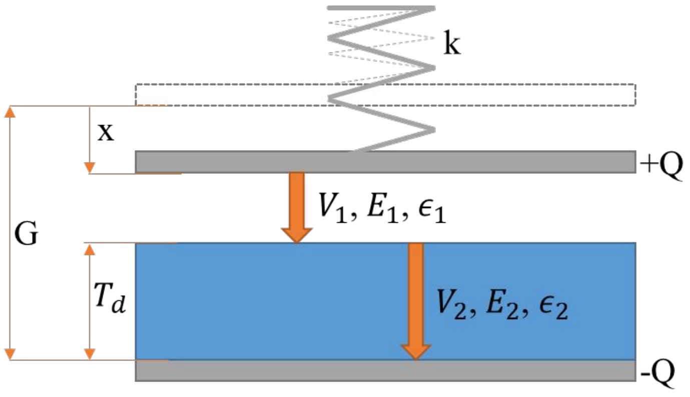

Figure S2 Cross section schematics of analytic model used for pull-in voltage estimation. Plate capacitor with movable top electrode and two in-series dielectric layers, where V, E, ϵ denote the voltage drop, electric field and relative permittivity of each layer.

The total voltage drop across the parallel plate capacitor can be split into the voltage drops across the dielectric layers:

$$V = V_1 + V_2 \tag{1}$$

Both V_1 and V_2 can be computed by integrating the electric fields inside the respective domains:

$$V_1 = \int_{x}^{g - t_d} E_1 \, dx$$
$$V_2 = \int_{g - t_d}^{g} E_2 \, dx \tag{2}$$

In general, the electric field within a plate capacitor is related to the charge as such:

$$E = \frac{Q}{\epsilon \, A} \tag{3}$$

where Q is the net charge on the plate, ϵ the permittivity of the medium between the electrodes, and A the electrode area.

Thus, we can rewrite equations 2 as:

$$V_1 = \int_x^{g-t_d} E_1\, dx = \int_x^{g-t_d} \frac{Q}{\epsilon_0\epsilon_1 A}\, dx = \frac{Q}{\epsilon_0\epsilon_1 A}(G - T_d - x)$$
$$V_2 = \int_{g-t_d}^{g} E_2\, dx = \int_{g-t_d}^{g} \frac{Q}{\epsilon_0\epsilon_2 A}\, dx = \frac{Q}{\epsilon_0\epsilon_2 A} T_d \tag{4}$$

Plugging equation 4 back into equation 1, we get the following expression for the total voltage drop:

$$V = \frac{Q}{\epsilon_0\epsilon_1 A}(G - T_d - x) + \frac{Q}{\epsilon_0\epsilon_2 A} T_d = \frac{Q}{A\epsilon_0\epsilon_1\epsilon_2}((G - T_d - x)\,\epsilon_2 + T_d\epsilon_1) = \frac{Q}{A\epsilon_0\epsilon_1}\left(G - T_d - x + \frac{T_d \epsilon_1}{\epsilon_2}\right) = \frac{Q}{A\epsilon_0\epsilon_1}\left(G - T_d\left(1 - \frac{\epsilon_1}{\epsilon_2}\right) - x\right) = \frac{Q}{A\epsilon_0\epsilon_1}(d - x) \tag{5}$$

where d was defined as $G - T_d\left(1 - \frac{\epsilon_1}{\epsilon_2}\right)$ and can be described as the geometric factor of the system.

We can express the total system capacitance $C = Q/V$ as:

$$C = \frac{Q}{\frac{Q}{A\epsilon_0\epsilon_1}(T - x)} = \frac{A\epsilon_0\epsilon_1}{d - x} \tag{6}$$

The pull-in condition is obtained when both mechanical and electrostatic forces are in equilibrium, and when the equivalent stiffness of the complete system becomes zero – any further displacement of the microbridge will not result in a restoring movement.

**Equilibrium**	$\boldsymbol{F_{total}} = \frac{\partial \boldsymbol{E_{el}}}{\partial \boldsymbol{x}} + \frac{\partial \boldsymbol{E_m}}{\partial \boldsymbol{x}} = \boldsymbol{0}$	(7)
**Equivalent Stiffness of complete system**	$\frac{\partial F_{total}}{\partial x} = \frac{\partial}{\partial x}(F_{el} + \mathrm{F_m}) = 0$	(8)

where $\boldsymbol{E_{el}}$ and $\boldsymbol{E_m}$ are the electrostatic, and mechanical energy, respectively:

$$E_{el} = \frac{1}{2} C\, V^2 \text{and } E_m = \frac{1}{2} k\, x^2$$

C: capacity, *V*: applied voltage, *k*: microbridge equivalent spring constant, *x*: displacement of microbridge

From equation 6 we get:

$$\boldsymbol{F_{total}} = \frac{\partial E_{el}}{\partial x} + \frac{\partial E_m}{\partial x} = \frac{\partial}{\partial x}\left(\frac{1}{2} C\, V^2\right) - \frac{\partial}{\partial x}\left(\frac{1}{2} k\, x^2\right) = \frac{\partial}{\partial x}\left(\frac{1}{2}\frac{A\epsilon_0\epsilon_1}{d - x} V^2\right) - k\, x = \frac{\epsilon_0\epsilon_1 A}{2(d - x)^2} V^2 - k\, x = 0 \tag{9}$$

Thus, we can compute the equilibrium voltage for a given spring constant:

$$V_{eq} = \sqrt{kx \frac{2(d - x)^2}{\epsilon_0\epsilon_1 A}} \tag{10}$$

Pull in is reached when the equivalent stiffness of the complete systems becomes zero. Thus, replacing the voltage with the equilibrium voltage from equation and computing the equivalent stiffness, the critical displacement at which pull-in occurs at:

$$\frac{\partial \boldsymbol{F_{total}}}{\partial \boldsymbol{x}} = \frac{\partial}{\partial \boldsymbol{x}}\left(\frac{\boldsymbol{\epsilon_0 \epsilon_1 A}}{\boldsymbol{2(d-x)^2}}\boldsymbol{V^2} - \boldsymbol{k\,x}\right) = -\boldsymbol{k} + \frac{\boldsymbol{AV^2 \epsilon_0 \epsilon_1}}{\boldsymbol{(d-x)^3}} = \boldsymbol{0} \tag{11}$$

Inserting $V = V_{eq}$

$$-\boldsymbol{k} + \frac{\boldsymbol{2kx}}{\boldsymbol{d-x}} = \boldsymbol{0} \tag{12}$$

Solving for x:

$$x_{PI} = \frac{d}{3} \tag{13}$$

Finally, the pull-in voltage can be calculated as:

$$V_{PI} = \sqrt{\frac{8kd^3}{27\,\epsilon_0 \epsilon_1 A}} \tag{14}$$

As for the equivalent spring constant of the microbridge, a clamped-clamped beam model with distributed uniform load is used:

$$k_0 = 32\,E\,W\left(\frac{T}{L}\right)^3 \tag{15}$$

The effect of the reduced area due to the holes on the electrostatic energy is said to be negligible when the diameter of the holes is less than 3-4 times the vertical gap between membrane and bottom electrode, which is the case here (300 nm diameter vs. 500nm total vertical gap). This is due to an increased fringing field effect that approximately compensates the loss of area.[2] However, the holes do influence the stiffness of the beam. Finite elements analysis shows that the equivalent spring constant is reduced by 45%.

$$k = k_0 \cdot 0.55 \tag{16}$$

The period-normalized hole diameter versus the equivalent spring constant (k) normalized by the one computed without the holes (k_0) for a beam of the given geometric and material properties (see Figure S1b) is plotted in Figure S3.

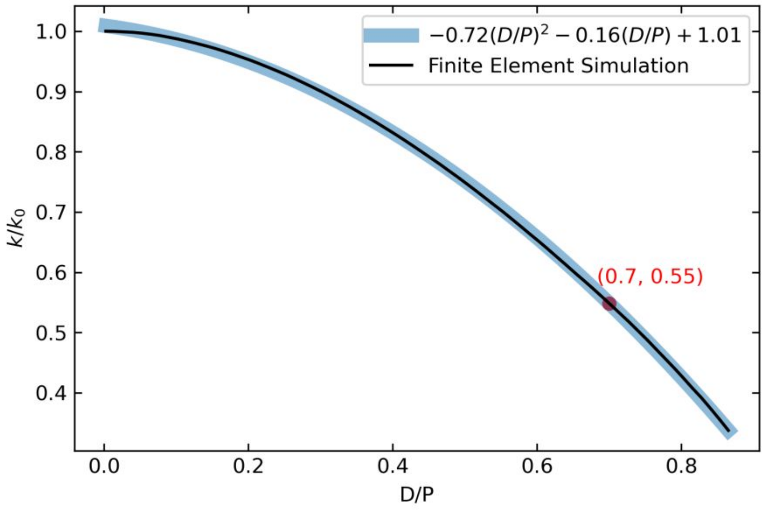

Figure S3 Influence of the hole diameter to period ratio on the equivalent spring constant. Simulation software: COMSOL Multiphysics

The computed pull-in voltage for the given structure was estimated to be **13.7 V**, which is in reasonable agreement with COMSOL Multiphysics simulations that predict a pull-in voltage of 16 V. The discrepancy between the two values can be attributed to the neglect of nonlinear effects in the analytical model, which are accounted for in the simulation. In fact, it can be shown that for a clamped-clamped beam, if the deflection is greater than half of the beam thickness, the tension along the beam cannot be ignored anymore[3]. Experimentally a pull-in voltage of approximately 25 V was observed. The discrepancy can be attributed to increased membrane rigidity resulting from residual stress-induced deformation (see Figure S5) as well as a discrepancy between the Young's modulus employed in simulation and that of the experimental device.

Mechanical Response Time

'The mechanical response time can be estimated by modelling the mechanical system as an undamped harmonic oscillator, and can be derived as follows:

Energy conservation: Gained Kinetic Energy + Remaining Potential Energy = Stored Potential Spring Energy

$$\frac{1}{2}m\dot{x}^2 + \frac{1}{2}mx^2 = \frac{1}{2}kd^2 \tag{5}$$

where *m* is the mass of the microbridge, *x* the instantaneous displacement, *k* the equivalent spring constant and *d* the maximum displacement of the microbridge.

The velocity is thus given by:

$$\dot{x} = \sqrt{\frac{k(d^2 - x^2)}{m}}. \tag{6}$$

Since $\dot{x} = dx/dt$, the latter can be rewritten to find the total time needed to travel from the actuated state back to the idle state:

$$t = \int_0^d \frac{dx}{\dot{x}} = \int_0^d \frac{dx}{\sqrt{\frac{k(d^2 - x^2)}{m}}} = \frac{\pi}{2}\sqrt{\frac{m}{k}} \tag{7}$$

Applying this equation to the present geometry (omitting the holes):

$$m = W\,L\,T\,\rho = 7.5\ \mu m \cdot 7.5\ um \cdot 100\ nm \cdot 2.285\frac{g}{cm^3} = 12.9 \cdot 10^{-12} g$$
$$\rightarrow t = 37.5\ ns. \tag{8}$$

Reflectivity dip in the idle state of the measured spectrum

The discrepancy between the initial simulated reflection spectrum and the measured reflection spectrum in the idle state can be explained by a geometry difference between the designed and effectively fabricated structures. As SEM images and digital holographic (DHM) measurements reveal (see Figure S5), the microbridge is buckled upwards in the idle state and its thickness is 80 nm instead of the designed 100 nm. Inputting these values into the RCWA simulation code reveals a closed match between the simulation and measured reflection spectra, as indicated in Figure S4.

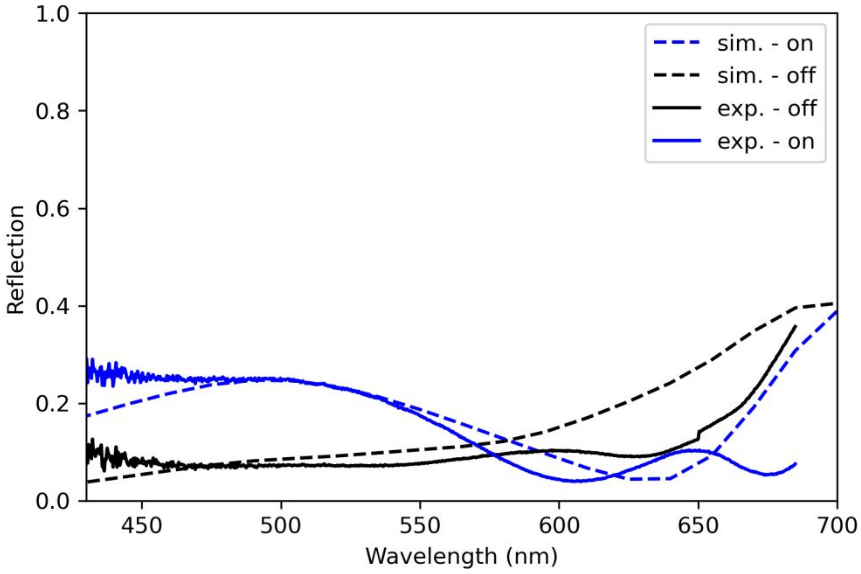

Figure S4: Simulated and experimental reflection spectrum. Geometry: 80nm buckled upwards, period: 400 nm, pillar diameter: 200 nm, trench: 50 nm, membrane thickness: 80 nm.

The reflectivity dip observed in the idle state of the measured spectrum – yielding the blue appearance of the pixels – can be explained by thin film destructive interference. Figure S5 visualizes the simplified analytic model used.

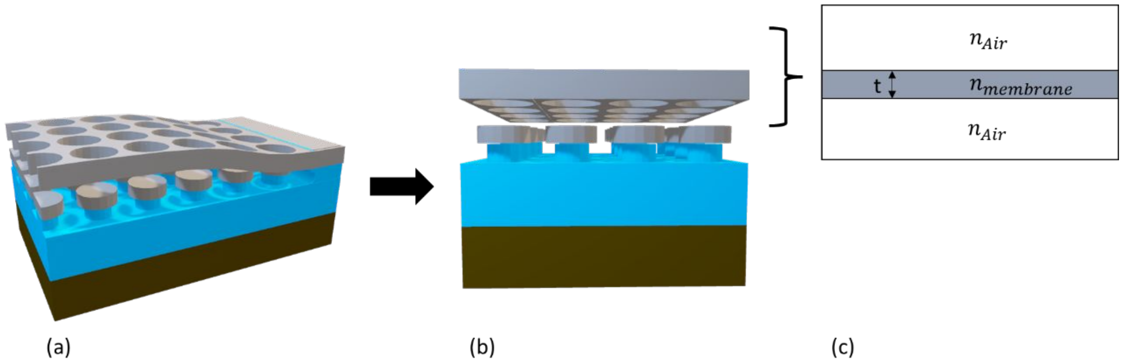

Figure S5: Visualization of the simplified model - The buckled membrane (a) is simplified to a membrane with constant offset from the pillar (b), and further simplified (c) to represent an infinite slab of thickness t and equivalent refractive index n_membrane.

Through digital holographic measurements it can be shown that the membrane is buckled upwards in the idle state (see Figure S5). Thus, most of its perforations are filled with air, leading to an approximate equivalent refractive index of 3 when taking the geometrical average:

$$n_{membrane} = n_{aSi}\frac{\left(P^2 - \frac{\pi}{4}H^2\right)}{A_{total}} + n_{Air}\frac{\frac{\pi}{4}H^2}{A_{total}} = 4.5\frac{400^2 - \frac{\pi}{4}300^2}{400^2} + 1\frac{\frac{\pi}{4}300^2}{400^2} = 2.95 \sim 3 \tag{9}$$

where P is the period and H the diameter of a hole in the microbridge.

In the following we will assume an infinite slab of refractive index 3, surrounded (top/bottom) by air. For this configuration ($n_{Air} < n_{Slab} < n_{Air}$), destructive interference is given when the thickness of the slab equals to:

$$t = \frac{m\,\lambda}{2\,n}\, for\; m = 1,2,3 \ldots \tag{10}$$

Thus, for m = 1, n = 3, and λ = [500 nm-700nm] a destructive interference is obtained when the thickness is between 83 nm – 117 nm. As the thickness of the microbridge is within these values, this is a strong indication as to why a reflectivity dip is seen in the (buckled) ON state.

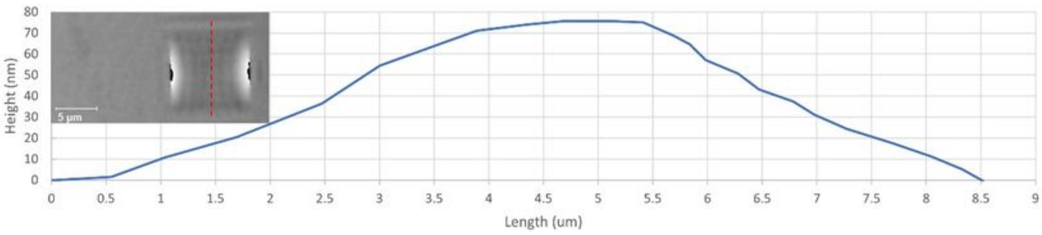

Figure S6 Digital Holographic Microscopy measurement of the buckled membrane. A maximum amplitude of approx. 80nm deflection is measured at the centre of the membrane. Red dotted line: measurement line.

Increasing the contrast ratio

The contrast ratio between idle and actuated state can be increased utilizing a more reflective membrane material, such as aluminum, as evidenced in the RCWA calculations reported in Figure S7.

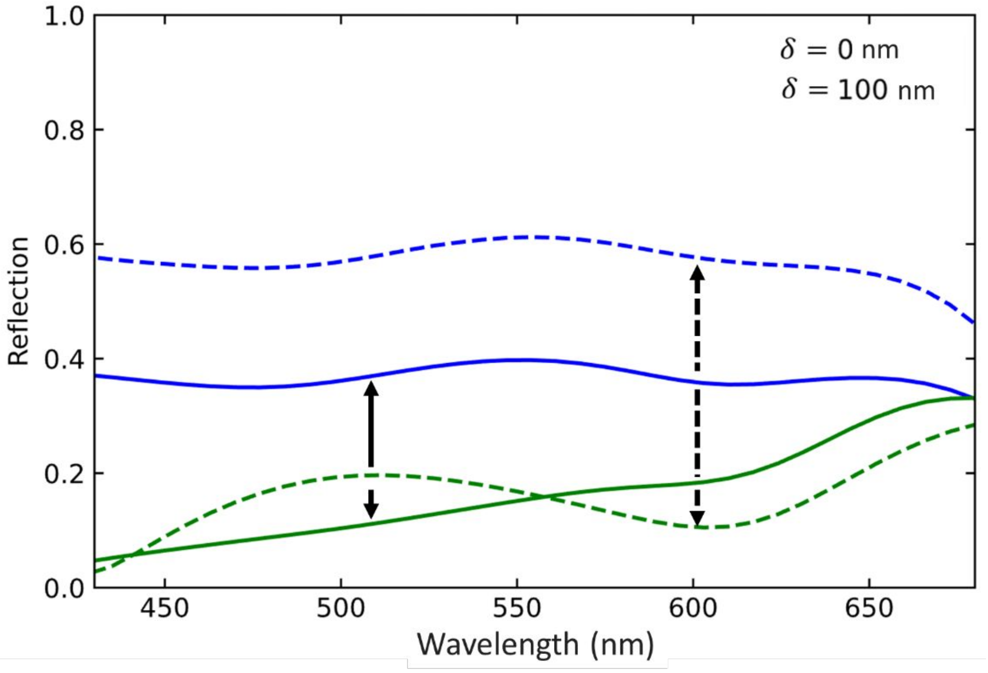

Figure S7: Influence of the membrane material on the reflection: Reflection spectra for different membrane positions δ = 0 nm (idle) and δ = 100 nm (actuated) and two different membrane materials: aSi (solid lines) and Al (dashed lines).

An increase in reflectivity beyond 0.6 could potentially be achieved by incorporating Al on the disks as well. However, in the actuated state, the absorption of Al-coated disks would be diminished, thereby adversely impacting the overall contrast. Thus, a compromise needs to be struck between increasing reflectivity and maintaining adequate contrast.

References:

(1) Saucedo-Flores, E.; Ruelas, R.; Flores, M.; Chiao, J. Study of the Pull-In Voltage for MEMS Parallel Plate Capacitor Actuators. *MRS Online Proceedings Library (OPL)* **2003**, *782*, A5.86.
(2) Rebeiz, G. M. *RF MEMS: Theory, Design, and Technology*; John Wiley & Sons, 2004.
(3) *What Is Geometric Nonlinearity?*. COMSOL. https://www.comsol.com/blogs/what-is-geometric-nonlinearity/ (accessed 2023-02-20).
